# Abnormal brain iron accumulation in obstructive sleep apnea: A quantitative MRI study in the HypnoLaus cohort

**DOI:** 10.1111/jsr.13698

**Published:** 2022-07-13

**Authors:** Nicola Andrea Marchi, Beatrice Pizzarotti, Geoffroy Solelhac, Mathieu Berger, José Haba‐Rubio, Martin Preisig, Peter Vollenweider, Pedro Marques‐Vidal, Antoine Lutti, Ferath Kherif, Raphael Heinzer, Bogdan Draganski

**Affiliations:** ^1^ Centre for Investigation and Research on Sleep, Department of Medicine Lausanne University Hospital (CHUV) and University of Lausanne Lausanne Switzerland; ^2^ Laboratory for Research in Neuroimaging, Department of Clinical Neurosciences Lausanne University Hospital (CHUV) and University of Lausanne Lausanne Switzerland; ^3^ Centre for Research in Psychiatric Epidemiology and Psychopathology, Department of Psychiatry Lausanne University Hospital (CHUV) and University of Lausanne Lausanne Switzerland; ^4^ Service of Internal Medicine, Department of Medicine Lausanne University Hospital (CHUV) and University of Lausanne Lausanne Switzerland; ^5^ Max Planck Institute for Human Cognitive and Brain Sciences Leipzig Germany

**Keywords:** ageing, Alzheimer's disease, neurodegeneration, R2*, sleep‐disordered breathing

## Abstract

Obstructive sleep apnea syndrome (OSA) may be a risk factor for Alzheimer's disease. One of the hallmarks of Alzheimer's disease is disturbed iron homeostasis leading to abnormal iron deposition in brain tissue. To date, there is no empirical evidence to support the hypothesis of altered brain iron homeostasis in patients with obstructive sleep apnea as well. Data were analysed from 773 participants in the HypnoLaus study (mean age 55.9 ± 10.3 years) who underwent polysomnography and brain MRI. Cross‐sectional associations were tested between OSA parameters and the MRI effective transverse relaxation rate (R2*) – indicative of iron content – in 68 grey matter regions, after adjustment for confounders. The group with severe OSA (apnea‐hypopnea index ≥30/h) had higher iron levels in the left superior frontal gyrus (*F*_3,760_ = 4.79, *p* = 0.003), left orbital gyri (*F*_3,760_ = 5.13, *p* = 0.002), right and left middle temporal gyrus (*F*_3,760_ = 4.41, *p* = 0.004 and *F*_3,760_ = 13.08, *p* < 0.001, respectively), left angular gyrus (*F*_3,760_ = 6.29, *p* = 0.001), left supramarginal gyrus (*F*_3,760_ = 4.98, *p* = 0.003), and right cuneus (*F*_3,760_ = 7.09, *p* < 0.001). The parameters of nocturnal hypoxaemia were all consistently associated with higher iron levels. Measures of sleep fragmentation had less consistent associations with iron content. This study provides the first evidence of increased brain iron levels in obstructive sleep apnea. The observed iron changes could reflect underlying neuropathological processes that appear to be driven primarily by hypoxaemic mechanisms.

## 1 INTRODUCTION

Obstructive sleep apnea (OSA) – a highly prevalent condition in the general population – is characterised by repetitive narrowing and/or collapse of the upper airways during sleep (Heinzer et al., 2015). Current evidence derived from clinical studies (Osorio et al., 2015; Yaffe et al., 2011) and meta‐analyses (Leng, McEvoy, Allen, & Yaffe, 2017; Zhu & Zhao, 2018) has identified obstructive sleep apnea as a risk factor for dementia, including Alzheimer's disease (AD) dementia. The hypothesis is that the key pathophysiological features of obstructive sleep apnea, namely hypoxaemia and sleep fragmentation, negatively impact brain health by increasing amyloid‐β deposition, inflammation, oxidative stress, and vascular dysregulation (Baril et al., 2018). Despite increasing awareness of the consequences of obstructive sleep apnea on brain health, our knowledge of the clinical features and putative biomarkers that put patients with obstructive sleep apnea at increased risk for dementia is still scarce (Gosselin, Baril, Osorio, Kaminska, & Carrier, 2019). The preclinical identification of “at risk” patients with obstructive sleep apnea may offer a window of opportunity for effective treatment to prevent neurodegeneration.

There is much controversy about the proper means to dissociate the unique contribution of ageing‐related disorders, including obstructive sleep apnea, from the “healthy” ageing effects on the brain. The current concept of “healthy” ageing accommodates a certain degree of cognitive decline and also a gradual accumulation of iron in the brain, the latter being linked to the progressive depletion of homeostatic iron mechanisms (Ward, Zucca, Duyn, Crichton, & Zecca, 2014). This is in contrast to neurodegenerative diseases that exhibit disturbed iron homeostasis that clearly exceeds the trajectories of “healthy” ageing (Ward et al., 2014). In the exemplary case of Alzheimer's disease, the abnormal degree of iron accumulation correlates with the amount of amyloid and tau pathology (van Duijn et al., 2017), as well as the rate of cognitive decline (Damulina et al., 2020). Therefore, published literature suggests that non‐invasive neuroimaging estimates of brain iron content can be used as biomarkers of neurodegeneration (Möller et al., 2019). However, to date, there is no empirical evidence to support the hypothesis of altered brain iron homeostasis in patients with obstructive sleep apnea.

Since the early 2000s, neuroimaging studies have described the association between obstructive sleep apnea and brain anatomical changes (Kumar et al., 2012; Macey et al., 2002; Macey et al., 2008; Morrell et al., 2003). However, despite advances in the field of neuroimaging, no consistent pattern of alterations in brain anatomy associated with OSA has emerged, even in recent large‐scale investigations (Marchi et al., 2020; Zuurbier et al., 2016). Besides differences in cohort sampling and computational anatomy methods, one plausible reason behind the controversy in the literature is the use of T1‐weighted imaging protocols that do not allow differentiation between atrophy related to a particular degenerative process and “spurious” morphometry differences due to pathology‐related MR signal changes (Lorio et al., 2016). Quantitative MRI, including relaxometry‐based mapping, hold promise for minimising these effects and reliably differentiating morphometry from brain tissue properties not only in the context of ageing (Draganski et al., 2011), but also in patients with obstructive sleep apnea (Marchi et al., 2020). Our established relaxometry MRI protocol is based on a biophysical model that allows a straightforward neurobiological interpretation of the MR signal sensitive to brain's tissue water, myelin, and iron content (Weiskopf, Mohammadi, Lutti, & Callaghan, 2015).

Based on our previous results (Marchi et al., 2020), we predicted that obstructive sleep apnea would be associated with increased iron content in grey matter regions known for high sensitivity to oxygen supply – medial temporal lobe, deep grey nuclei, and cortical watershed areas (Marchi et al., 2020). We tested our hypothesis in a large‐scale community‐dwelling cohort using objective measures of obstructive sleep apnea derived from polysomnography (PSG) and quantitative MRI relaxometry estimates of brain iron content.

## 2 METHODS

### 2.1 Sample

Participants were selected from the longitudinal community‐dwelling cohort CoLaus|PsyCoLaus that included regular physical and psychiatric evaluations (Firmann et al., 2008). A total of 6734 individuals aged 35–75 years were randomly selected from residents of the city of Lausanne (Switzerland) between 2003 and 2006 according to the civil registry. Three follow‐up evaluations took place after the baseline assessment. For the present study, we included 773 participants who underwent a full‐night polysomnography at home (HypnoLaus) and a brain MRI (BrainLaus), as reported previously (Marchi et al., 2020) (Figure S1). All participants gave their written informed consent before starting the study. The local institutional review board approved the CoLaus|PsyCoLaus, HypnoLaus, and BrainLaus studies.

### 2.2 Polysomnographic data

Polysomnography data were recorded as part of the nested HypnoLaus study performed between 2009 and 2013 (Heinzer et al., 2015). The methodology for PSG recording has been described previously (Heinzer et al., 2015). Briefly, sleep technicians visually scored PSG recordings using Somnologica software (version 5.1.1, Embla Flaga). Sleep stages and arousals were scored according to the 2007 American Academy of Sleep Medicine (AASM) manual. Respiratory events were scored according to the 2012 AASM manual. We defined apnea as a ≥90% drop of airflow from baseline lasting ≥10 s. We defined hypopnea as a ≥30% drop of airflow lasting ≥10 s with either an arousal or a ≥3% oxygen desaturation. The apnea‐hypopnea index (AHI) was calculated as the average number of apneas and hypopneas per hour of sleep. The sample was categorised into four groups according to AHI: no OSA (0–4.9 events/h), mild OSA (5.0–14.9 events/h), moderate OSA (15.0–29.9 events/h), and severe OSA (≥30 events/h). Participants who met the criteria for central sleep apnea syndromes were excluded (*n* = 2) (American Academy of Sleep Medicine. International classification of sleep disorders, 3rd ed, 2014). The following sleep parameters were also used in the analyses: total sleep time (TST), sleep efficiency, arousal index (defined as the number of arousal per hour of sleep), proportion of sleep stages N1, N2, N3, and rapid eye movement (REM), 3% and 4% oxygen desaturation index (ODI‐3 and ODI‐4, respectively), proportion of TST spent with oxygen saturation <90% (T90) and hypoxic load (defined as the total area under the curve of any ≥3% oxygen desaturations related to an apnea or hypopnea event and divided by the TST) (Berger et al., 2020).

### 2.3 Brain **MRI** data

Neuroimaging data from the nested BrainLaus study collected between 2014 and 2018 were analysed (Trofimova et al., 2021). Participants had an MRI on average 4.4 ± 1.0 years after the polysomnography. Data were acquired on a 3T whole‐body MRI system (Magnetom Prisma, Siemens Medical Systems, Germany), using a 64‐channel radiofrequency (RF) receive head coil and body coil for transmission. The quantitative MRI protocol included three multi‐echo 3D fast low angle shot (FLASH) acquisitions with magnetisation transfer‐weighted (MTw: repetition time [TR] = 24.5 ms, *α* = 6°), proton density‐weighted (PDw: TR = 24.5 ms, *α* = 6°), and T1‐weighted (TR = 24.5 ms, *α* = 21°) contrasts at 1 mm isotropic resolution (Weiskopf et al., 2013). Echo images were acquired for the MTw, PDw, and T1w contrasts (minimal echo time: 2.34 ms; inter‐echo spacing: 2.34 ms) (Lutti et al., 2022). The smaller number of echo images for the MTw contrast was the result of the RF pulse used for MT saturation (4 ms duration, 220° nominal flip angle, 2 kHz frequency offset from water resonance). To correct for the effects of RF transmit field inhomogeneities on the quantitative MRI maps (Lutti, Dick, Sereno, & Weiskopf, 2014), B1 mapping data were acquired using the 3D echo‐planar imaging (EPI) spin‐echo and stimulated echo method as previously described (4 mm^3^ resolution, echo time [TE] = 39.06 ms, TR = 500 ms) (Lutti et al., 2012; Lutti, Hutton, Finsterbusch, Helms, & Weiskopf, 2010). B0‐field mapping data were acquired to correct for image distortions in the EPI data (2D double‐echo FLASH sequence with slice thickness = 2 mm, TR = 1020 ms, TE1/TE2 = 10/12.46 ms, *α* = 90°, bandwidth = 260 Hz/pixel). The total acquisition time was 27 min. Quantitative MRI maps of MT, R1, PD, and R2* were calculated offline from the raw data as described previously (Helms, Dathe, & Dechent, 2008; Helms, Dathe, Kallenberg, & Dechent, 2008), in the framework of SPM12 (www.fil.ion.ucl.ac.uk/spm; Wellcome Centre for Human Neuroimaging) using customised MATLAB tools (The Mathworks). In particular, the effective transverse relaxation rate R2* was calculated from the raw FLASH images using regression of the log signal with the corresponding echo times) (Callaghan et al., 2014; Castella et al., 2018). The present study focussed only on R2* data as estimates of brain tissue iron. All 22 raw echo images from the MTw, T1w and PDw contrasts were used for estimation of R2* to maximise fitting robustness (Weiskopf, Callaghan, Josephs, Lutti, & Mohammadi, 2014). In individual's native space regional averages of R2* were extracted from areas defined by factorisation‐based image labelling (Yan, Balbastre, Brudfors, & Ashburner, 2021). R2* data from 68 grey matter regions covering most cortical and subcortical brain areas were analysed. R2* values are presented as s^−1^.

### 2.4 Demographic and clinical variables

Our statistical analyses included demographic and clinical variables assessed at the time of polysomnography, except for continuous positive airway pressure therapy (CPAP) (see below). The body mass index (BMI) was calculated as weight/height^2^. The presence of diabetes was defined as a fasting blood glucose ≥7 mmol/L and/or antidiabetic drug use. Hypertension was defined as a systolic blood pressure ≥140 mmHg and/or diastolic blood pressure ≥90 mmHg and/or antihypertensive drug use. Dyslipidaemia was determined by a low high‐density lipoprotein cholesterol (<1.0 mmol/L) and/or high triglyceride (≥2.2 mmol) and/or high low‐density lipoprotein cholesterol (≥4.1 or ≥2.6 mmol/L in the presence of self‐reported history of myocardial infarction, stroke, coronary artery disease, or diabetes) and/or lipid‐lowering treatment. Smoking habits were categorised in (ex‐)smoker or never smoked. Alcohol consumption was dichotomised into ≥14 or <14 units/week. While there were no CPAP‐treated participants at the time of polysomnography, 27 participants (3.5%) initiated CPAP treatment between PSG and MRI data acquisition.

### 2.5 Statistical analysis

Descriptive statistics examined the differences between OSA groups using the chi‐square or Fisher's exact test for categorical variables and analysis of variance or Kruskal‐Wallis test for continuous variables. We tested for differences in R2* between OSA groups using analysis of covariance (ANCOVA). To test the association between R2* and sleep parameters in the whole sample, we performed linear regression analyses with R2* as dependent variable and individual sleep parameters as independent variables. Sleep parameters were tested for normal distribution using Q–Q plot. In case of a skewed distribution, the variable was log‐transformed and, if skewed distribution persisted, the variable was categorised into quartiles. Finally, four variables were log‐transformed – arousal index, AHI, ODI‐3, and hypoxic load, and three variables were categorised into quartiles – sleep efficiency, ODI‐4, and T90. ANCOVA and linear regression models were both corrected for age, sex, BMI, diabetes, dyslipidaemia, hypertension, and CPAP given that these variables showed significant differences between OSA groups. Time interval between PSG and MRI and smoking (a determinant of brain iron accumulation) (Pirpamer et al., 2016) were also included in the models. Regarding ANCOVA, continuous variables were included as covariates in the model (age, time between PSG and MRI, and BMI) and categorical variables as fixed factors (sex [male/female], diabetes [yes/no], dyslipidaemia [yes/no], hypertension [yes/no], smoking [yes/no], and CPAP [yes/no]). All statistical analyses were tested for violations of the model assumptions and any conflicts and resolutions are reported. To account for multiple testing, we set the statistical significance level at a value of *p* <0.005, according to recent recommendations (Benjamin et al., 2018; Ioannidis, 2018). For statistical analysis, we used IBM SPSS Statistics Version 27.0.

## 3 RESULTS

### 3.1 Participant characteristics

The mean age of the sample was 55.9 ± 10.3 years (range: 40–84 years) with almost equal representation of men and women. The number of participants with mild, moderate, and severe obstructive sleep apnea was 286 (37.0%), 164 (21.2%), and 82 (10.6%), respectively. Detailed characteristics of the sample are presented in Table 1.

**TABLE 1:** Sample characteristics. Values are presented as the number of participants (%) for categorical variables and mean ± standard deviation or median [range] for continuous variables. Variables were recorded at the time of polysomnography, except for continuous positive airway pressure treatment that was recorded at MRI visit

	All sample	No OSA	Mild OSA	Moderate OSA	Severe OSA	*P* value
	*n* = 773 (100%)	*n* = 241 (31.2%)	*n* = 286 (37.0%)	*n* = 164 (21.2%)	*n* = 82 (10.6%)	
Age, years	55.9 ± 10.3	52.8 ± 9.7	55.9 ± 9.9	58.0 ± 10.1	60.7 ± 10.9	**<0.001**
Male, no. (%)	383 (49.5)	63 (26.1)	138 (48.3)	116 (70.7)	66 (80.5)	**<0.001**
BMI, kg/m^2^	25.6 ± 4.0	23.7 ± 3.5	25.6 ± 3.8	26.9 ± 3.9	28.1 ± 3.6	**<0.001**
Diabetes, no. (%)	51 (6.6)	7 (2.9)	13 (4.5)	20 (12.2)	11 (13.4)	**<0.001**
Dyslipidaemia, no. (%)	339 (43.9)	72 (29.9)	124 (43.4)	92 (56.1)	51 (62.2)	**<0.001**
Hypertension, no. (%)	247 (32.0)	45 (18.7)	91 (31.8)	66 (40.2)	45 (54.9)	**<0.001**
Smoking, no. (%)	427 (55.2)	125 (51.9)	157 (54.9)	99 (60.4)	46 (56.1)	0.409
Alcohol, no. (%)	119 (15.4)	32 (13.3)	42 (14.7)	31 (18.9)	14 (17.1)	0.446
CPAP, no. (%)	27 (3.5)	0 (0.0)	2 (0.7)	12 (7.3)	13 (15.9)	**<0.001**
Total sleep time, min	401.7 ± 73.1	408.5 ± 67.7	403.9 ± 77.8	399.6 ± 69.7	378.3 ± 393.5	0.012
Sleep efficiency, %	89.5 [11.8–99.1]	90.5 [45.8–98.9]	90.5 [11.8–99.1]	88.0 [46.4–98.0]	82.9 [29.6–97.7]	**<0.001**
Arousal index, no./h	18.1 [2.4–70.9]	14.5 [2.4–53.4]	17.2 [4.7–55.8]	21.3 [7.8–49.5]	32.3 [13.1–70.9]	**<0.001**
N1 stage, %	11.5 ± 6.8	9.7 ± 4.6	10.5 ± 5.7	13.2 ± 7.4	16.9 ± 10.1	**<0.001**
N2 stage, %	45.3 ± 9.8	45.2 ± 8.9	45.2 ± 9.5	45.2 ± 10.3	46.6 ± 12.3	0.703
N3 stage, %	20.7 ± 8.3	22.3 ± 7.8	21.4 ± 8.3	18.9 ± 8.3	17.5 ± 8.0	**<0.001**
REM sleep, %	22.5 ± 6.2	22.8 ± 5.5	22.9 ± 6.2	22.8 ± 6.0	19.1 ± 7.5	**<0.001**
AHI, no./h	8.6 [0.0–82.9]	2.3 [0.0–4.9]	8.6 [5.0–14.9]	20.0 [15.0–29.9]	41.2 [30.1–82.9]	**<0.001**
ODI‐3, no./h	8.7 [0.0–79.6]	2.3 [0.0–18.0]	8.4 [1.2–22.5]	18.2 [5.6–44.6]	33.9 [19.9–79.6]	**<0.001**
ODI‐4, no./h	3.1 [0.0–62.3]	0.4 [0.0–7.0]	3.0 [0.0–11.4]	9.6 [1.7–24.3]	22.4 [10.2–62.3]	**<0.001**
T90, %	0.1 [0.0–100.0]	0.0 [0.0–100.0]	0.1 [0.0–66.7]	0.7 [0.0–62.0]	3.4 [0.0–99.9]	**<0.001**
Hypoxic load, (%min)/h	3.4 [0.0–159.6]	1.0 [0.0–8.3]	4.3 [0.0–19.4]	12.5 [1.3–39.1]	36.8 [1.2–159.6]	**<0.001**

*Note*: Bold text indicates *p* <0.005.

Abbreviations: AHI, apnea‐hypopnea index; BMI, body mass index; CPAP, continuous airway positive pressure; ODI, oxygen desaturation index; OSA, obstructive sleep apnea; T90, percentage of sleep time with oxygen saturation <90%.

### 3.2 **R2** * signal analysis according to **OSA** groups

Differences were observed in the R2* signal between groups in the left superior frontal gyrus (*F*_3,760_ = 4.79, *p* = 0.003), left orbital gyri (*F*_3,760_ = 5.13, *p* = 0.002), right and left middle temporal gyrus (*F*_3,760_ = 4.41, *p* = 0.004 and *F*_3,760_ = 13.08, *p* < 0.001, respectively), left angular gyrus (*F*_3,760_ = 6.29, *p* = 0.001), left supramarginal gyrus (*F*_3,760_ = 4.98, *p* = 0.003), and right cuneus (*F*_3,760_ = 7.09, *p* < 0.001). In the right cuneus, post hoc tests showed higher R2* in the severe OSA group compared with the no OSA and mild OSA groups and also higher R2* in the moderate OSA group compared with the no OSA group (*p* <0.05, after Bonferroni correction for multiple comparisons). For the other brain regions, post hoc tests showed higher R2* in the severe OSA group compared with the other three groups (Table 2 and Table S1).

**TABLE 2:** Significant differences in R2* among obstructive sleep apnea groups. Findings are s^−1^ and are presented as adjusted mean ± standard error. Data were analysed by analysis of covariance using R2* as the dependent variable and obstructive sleep apnea groups as independent variable. Models are adjusted for age, time between polysomnography and MRI, sex, body mass index, diabetes, dyslipidaemia, hypertension, smoking, and continuous positive airway pressure treatment

	No OSA	Mild OSA	Moderate OSA	Severe OSA	*p*
	*n* = 241 (31.2%)	*n* = 286 (37.0%)	*n* = 164 (21.2%)	*n* = 82 (10.6%)	
L superior frontal gyrus	17.91 ± 0.12	17.95 ± 0.12	17.94 ± 0.12	18.36 ± 0.13^a^ ^,^ ^b^ ^,^ ^c^	**0.003**
L orbital gyri	17.44 ± 0.10	17.50 ± 0.10	17.43 ± 0.10	17.81 ± 0.11^a^ ^,^ ^b^ ^,^ ^c^	**0.002**
R middle temporal gyrus	17.88 ± 0.12	18.02 ± 0.12	18.02 ± 0.12	18.35 ± 0.14^a^ ^,^ ^b^ ^,^ ^c^	**0.004**
L middle temporal gyrus	21.22 ± 0.20	21.44 ± 0.19	21.54 ± 0.19	22.15 ± 0.22^a^ ^,^ ^b^ ^,^ ^c^	**<0.001**
L angular gyrus	19.60 ± 0.15	19.78 ± 0.15	19.77 ± 0.15	20.24 ± 0.17^a^ ^,^ ^b^ ^,^ ^c^	**0.001**
L supramarginal gyrus	19.94 ± 0.14	20.09 ± 0.14	20.07 ± 0.14	20.50 ± 0.16^a^ ^,^ ^b^ ^,^ ^c^	**0.003**
R cuneus	20.19 ± 0.14	20.36 ± 0.14	20.55 ± 0.14^a^	20.84 ± 0.16^a^ ^,^ ^b^	**<0.001**

*Note*: Bold text indicates *p* <0.005.

Abbreviations: L, left; R, right.

^a^ Significant difference compared with no OSA group.

^b^ Significant difference compared with mild OSA group.

^c^ Significant difference compared with moderate OSA group.

### 3.3 Associations between **R2** * signal and **PSG** parameters

**FIGURE 1:**
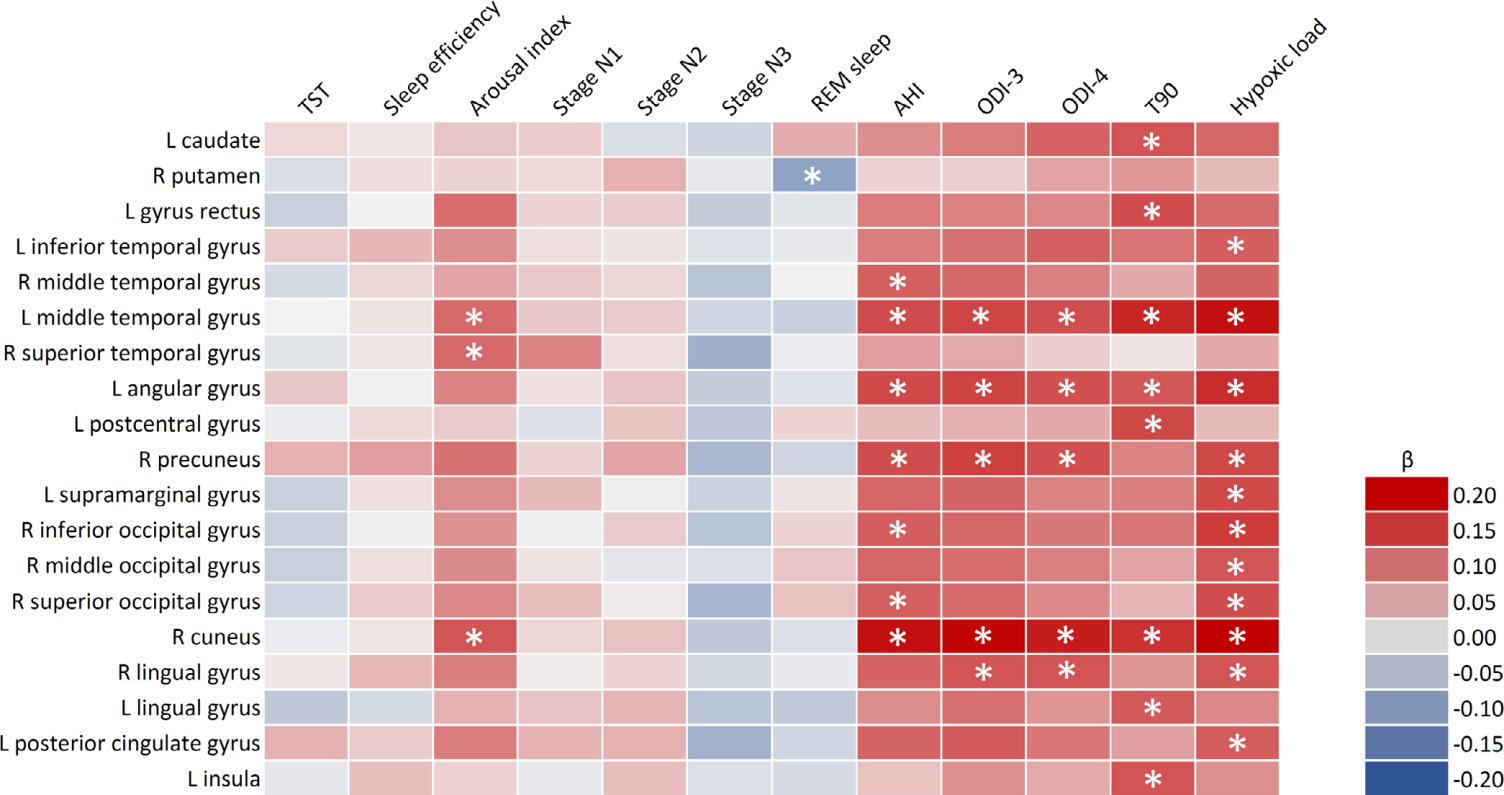
Heatmap of the significant associations between R2* and sleep variables. Data were analysed by linear regression using R2* as the dependent variable and individual sleep variables as independent variables. Models are adjusted for age, time between polysomnography and MRI, sex, body mass index, diabetes, dyslipidaemia, hypertension, smoking, and continuous positive airway pressure treatment. Asterisks indicate *p* <0.005. AHI, apnea‐hypopnea index; L, left; ODI, oxygen desaturation index; R, right; T90, percentage of sleep time with oxygen saturation <90%; TST, total sleep time; β, standardised beta coefficient

A higher arousal index was associated with higher R2* in the left middle temporal gyrus, right superior temporal gyrus, and right cuneus. A lower proportion of REM sleep was associated with higher R2* in the right putamen. A higher AHI was linked to higher R2* in the bilateral middle temporal gyrus, left angular gyrus, right precuneus, right inferior occipital gyrus, right superior occipital gyrus, and right cuneus. Higher ODI‐3 and ODI‐4 were both associated with higher R2* in the left middle temporal gyrus, left angular gyrus, right precuneus, right cuneus, and right lingual gyrus. A higher T90 was linked to higher R2* in the left caudate, left gyrus rectus, left middle temporal gyrus, left angular gyrus, left postcentral gyrus, right cuneus, left lingual gyrus, and left insula. A higher hypoxic load was related to higher R2* in the left inferior temporal gyrus, left middle temporal gyrus, left angular gyrus, right precuneus, left supramarginal gyrus, right inferior occipital gyrus, right middle occipital gyrus, right superior occipital gyrus, right cuneus, right lingual gyrus, and left posterior cingulate gyrus. The findings are summarised in Figure 1 (for more details, see Figure S2, Tables S1 and S2).

## 4 DISCUSSION

Community‐dwelling individuals with severe obstructive sleep apnea had a higher R2* in specific cortical regions of the frontal (left orbital gyri and left superior frontal gyrus), temporal (bilateral middle temporal gyrus), parietal (left angular gyrus and left supramarginal gyrus), and occipital lobes (right cuneus), which we interpreted as increased iron accumulation. Nocturnal hypoxaemia appeared to be the primary driver of iron alterations given the consistent association between all hypoxaemia variables and increased iron content. This contrasted with measures of sleep fragmentation, which showed less pronounced associations with iron content. We see two main implications of our study – first, it provides additional evidence of the negative impact of obstructive sleep apnea on brain's anatomy by going beyond established metrics of volume and cortical thickness; second, given the evidence for “spurious” morphometric results associated with microstructural tissue changes (including iron changes) (Lorio et al., 2016), it offers a potential explanation for some of the controversial findings reported in the literature.

The topological pattern of iron changes in the group with severe obstructive sleep apnea included the prefrontal cortex, middle temporal gyrus, inferior parietal lobule, and cuneus. This spatial pattern corroborates the results of previous neuroimaging studies showing OSA‐associated morphometric alterations in these areas (Shi et al., 2017; Weng et al., 2014), which correlated with the degree of nocturnal hypoxaemia in some studies (Marchi et al., 2020; Zuurbier et al., 2016). Furthermore, a recent study – which used a multimodal neuroimaging approach – revealed an increased amyloid burden, morphometric changes, altered perfusion, and altered glucose metabolism overlapping in the medial parieto‐occipital lobe, including the cuneus, in the OSA group compared with controls (André et al., 2020). Most of these regions have consistently shown early morphometric changes in mild cognitive impairment and Alzheimer's disease and represent key nodes in the networks of cognitive functions supporting attention, working memory, spatial cognition, language, and number processing (Long, 2019).

Our hypothesis was only partially confirmed because we also expected consistent associations between obstructive sleep apnea parameters and iron content in the hippocampus‐amygdala complex and deep grey nuclei. Concerning deep grey nuclei, we only found associations between a lower proportion of REM sleep and a higher iron content in the right putamen, and between T90 and higher iron content in the left caudate. This suggests a complex relationship between obstructive sleep apnea, macrostructural morphometric alterations (i.e., changes in volume and cortical thickness), and changes in iron content, in which macrostructural alterations do not necessarily appear to be associated with changes in iron content and *vice versa*. Besides the technical consideration of neuroimaging mentioned above (Lorio et al., 2016), this complex relationship could be also explained by the fact that iron is heterogeneously distributed in the brain and its regulation appears to be closely dependent on local homeostatic processes (Möller et al., 2019; Ward et al., 2014).

To our knowledge, no previous neuroimaging or postmortem study has provided results on the association between obstructive sleep apnea and iron in brain tissue in humans. Only a few recent mice investigations have reported on increased iron accumulation in the hippocampus after exposure to an intermittent hypoxia protocol (An et al., 2020; Zhao et al., 2021). Therefore, it is difficult to speculate on the casual link between obstructive sleep apnea and iron changes, but some hypotheses could be formulated. Given the association between obstructive sleep apnea and Alzheimer's disease, the observed increase in iron content may reflect deposition of amyloid‐β and/or tau protein – especially in the medial parieto‐occipital lobe (André et al., 2020) – as iron is abundantly present in amyloid plaques and neurofibrillary tangles (Smith, Harris, Sayre, & Perry, 1997; van Duijn et al., 2017). A second hypothesis is that obstructive sleep apnea and related features (i.e., nocturnal hypoxaemia and/or sleep fragmentation) may promote inflammatory processes and the recruitment of iron‐accumulating microglia, which has been shown to contribute to increased brain iron load in neurodegeneration (Kenkhuis et al., 2021; van Duijn et al., 2017). Our third hypothesis is about a negative effect of obstructive sleep apnea on myelin integrity that may induce remyelination processes driven by oligodendrocytes, which are the predominant iron‐containing cells in the brain (Connor & Menzies, 1996; van Duijn et al., 2017). All these hypotheses need further investigations.

From a clinical perspective, this study suggests the usefulness of imaging biomarkers – sensitive to brain tissue properties – to detect OSA‐induced pathology (Möller et al., 2019). The relaxometry‐based R2* protocol is potentially available for clinical MRI systems, can provide artefact‐free and high‐resolution images in short time, and does not require extensive post‐processing analysis (Langkammer, Ropele, Pirpamer, Fazekas, & Schmidt, 2014). Given that the brain iron content has been shown to correlated with cognitive performance in the general population (Penke et al., 2012) and in Alzheimer's disease (Damulina et al., 2020), future studies should examine this relationship in obstructive sleep apnea. Indeed, the investigation of potential neuroimaging biomarkers for the identification of OSA patients at risk of cognitive decline is of clinical interest, as these biomarkers could be useful for improving therapeutic decision making.

The strengths of our study were the use of a large and well‐characterised cohort, polysomnographic measures of obstructive sleep apnea, and the estimation of brain iron content using a well‐established MRI relaxometry protocol. Limitations are the cross‐sectional design and the time gap between polysomnography and MRI, although our analyses were adjusted for this parameter. Furthermore, obstructive sleep apnea is a stable condition that tends to increase slightly with age but rarely shows important severity variations unless in the presence of substantial weight changes. In our sample, the average BMI difference between PSG and MRI visits was negligible (0.4 ± 1.6 kg/m^2^) and including this parameter as a confounder in the analyses did not show any significant impact on our findings (data not shown). Iron has been shown to be the major determinant of R2* in grey matter, but we cannot completely exclude the possibility that confounding factors, such as increased myelination, calcifications, or blood depositions, also contributed to the increased R2* values (Weiskopf et al., 2015). However, myelination does not increase in middle age and older adulthood, and we do not expect participants with obstructive sleep apnea to have increased intracranial calcifications or blood deposits. R2* is sensitive to differences in blood oxygenation, given the differential magnetic susceptibility of oxyhaemoglobin and deoxyhaemoglobin. However, the MRI scans were performed during wakefulness, whereas the intermittent oxygen desaturation observed in obstructive sleep apnea occurs during sleep. Therefore, it is unlikely that OSA‐related desaturation events would alter the R2* signal during image acquisition.

In conclusion, we provided the first evidence for an association between obstructive sleep apnea and increased brain iron levels, which could reflect underlying neuropathological processes. Future studies should examine whether neuroimaging biomarkers of iron content could be useful in identifying obstructive sleep apnea patients at increased risk for cognitive decline.

## AUTHOR CONTRIBUTIONS

Conception and design of the study: N.A.M., R.H., and B.D. Acquisition and analysis of data: N.A.M., G.S., M.B., A.L., F.K., M.P., P.M.V., P.V., R.H., and B.D. Drafting a significant portion of the manuscript or figures: N.A.M., B.P., R.H., and B.D.

## CONFLICT OF INTEREST

R.H. reports having received consulting or speaking fees from Resmed, Inspire, Philips, Bioprojet, and Jazz Medical. Non‐financial Disclosure: R.H. reports being member of the medical advisory boards of Dreem, NightBalance, and Philips. All remaining authors report no conflicts of interest.

## Supporting information


**APPENDIX S1** Supporting Information.

## Data Availability

Due to the sensitivity of the data and the lack of consent for online posting, individual data cannot be made accessible. Only metadata will be made available in digital repositories. Metadata requests can also be performed via the study website www.colaus-psycolaus.ch.
