# Supplementary material for: Abnormal brain iron accumulation in obstructive sleep apnea: A quantitative MRI study in the HypnoLaus cohort
Source: J Sleep Res. 2022 Jul 13;31(6):e13698. doi: 10.1111/jsr.13698 (PMC9787990; doi:10.1111/jsr.13698)
Supplement: Supplementary file 1 — APPENDIX S1 Supporting Information. [file JSR-31-e13698-s001.docx]

**SUPPORTING INFORMATION**

**Abnormal brain iron accumulation in obstructive sleep apnea: a quantitative MRI study in the HypnoLaus cohort**

Nicola Andrea Marchi, MD,^1,2^ Beatrice Pizzarotti, MD,^2^ Geoffroy Solelhac, MD,^1^ Mathieu Berger, PhD,^1^ José Haba-Rubio, MD,^1^ Martin Preisig, MD,^3^ Peter Vollenweider, MD,^4^ Pedro Marques-Vidal, MD, PhD,^4^ Antoine Lutti, PhD,^2^ Ferath Kherif, PhD,^2^ Raphael Heinzer, MD, MPH,^1,^* and Bogdan Draganski, MD^2,5,^*

*^1^Center for Investigation and Research on Sleep, Department of Medicine, Lausanne University Hospital (CHUV) and University of Lausanne, Lausanne, Switzerland.*

*^2^Laboratory for Research in Neuroimaging, Department of Clinical Neurosciences, Lausanne University Hospital (CHUV) and University of Lausanne, Lausanne, Switzerland.*

*^3^Center for Research in Psychiatric Epidemiology and Psychopathology, Department of Psychiatry, Lausanne University Hospital (CHUV) and University of Lausanne, Lausanne, Switzerland.*

*^4^Service of Internal Medicine, Department of Medicine, Lausanne University Hospital (CHUV) and University of Lausanne, Lausanne, Switzerland.*

*^5^Max Planck Institute for Human Cognitive and Brain Sciences, Leipzig, Germany.*

**These authors contributed equally to this work as senior authors.*

**Supplementary Figure 1. Flowchart of the study population.**

**
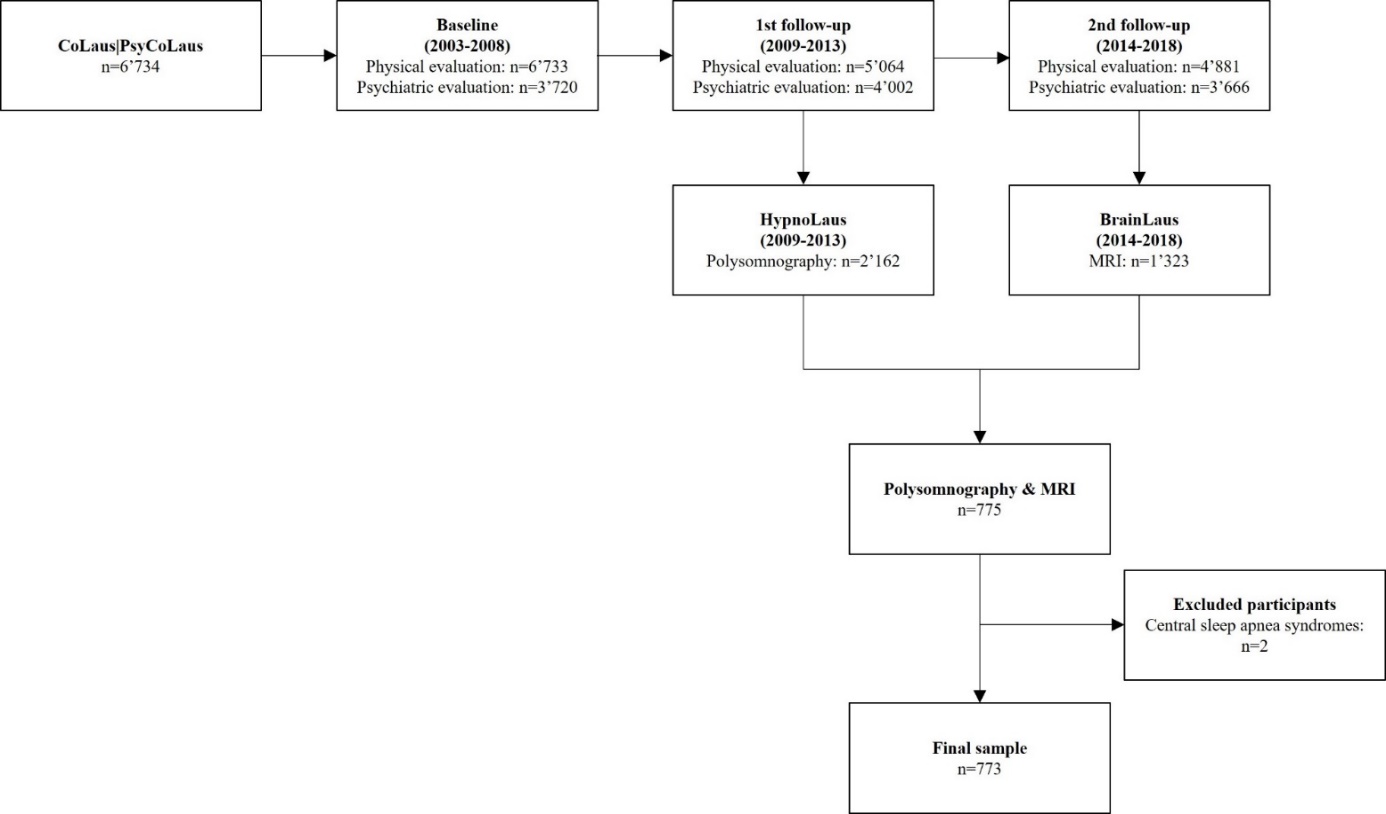
**

**Supplementary Figure 2. Heatmap of the associations between R2* and sleep variables.** Data are analyzed by linear regression using R2* as dependent variable and individual sleep variables as independent variables. Models are adjusted for age, time between polysomnography and MRI, sex, body mass index, diabetes, dyslipidemia, hypertension, smoking, and continuous positive airway pressure treatment. Asterisks indicate p-value < 0.005. Abbreviations: AHI = apnea-hypopnea index; L = left; ODI = oxygen desaturation index; R = right; T90 = percentage of sleep time with oxygen saturation < 90%; TST = total sleep time; β = standardized beta coefficient.

**
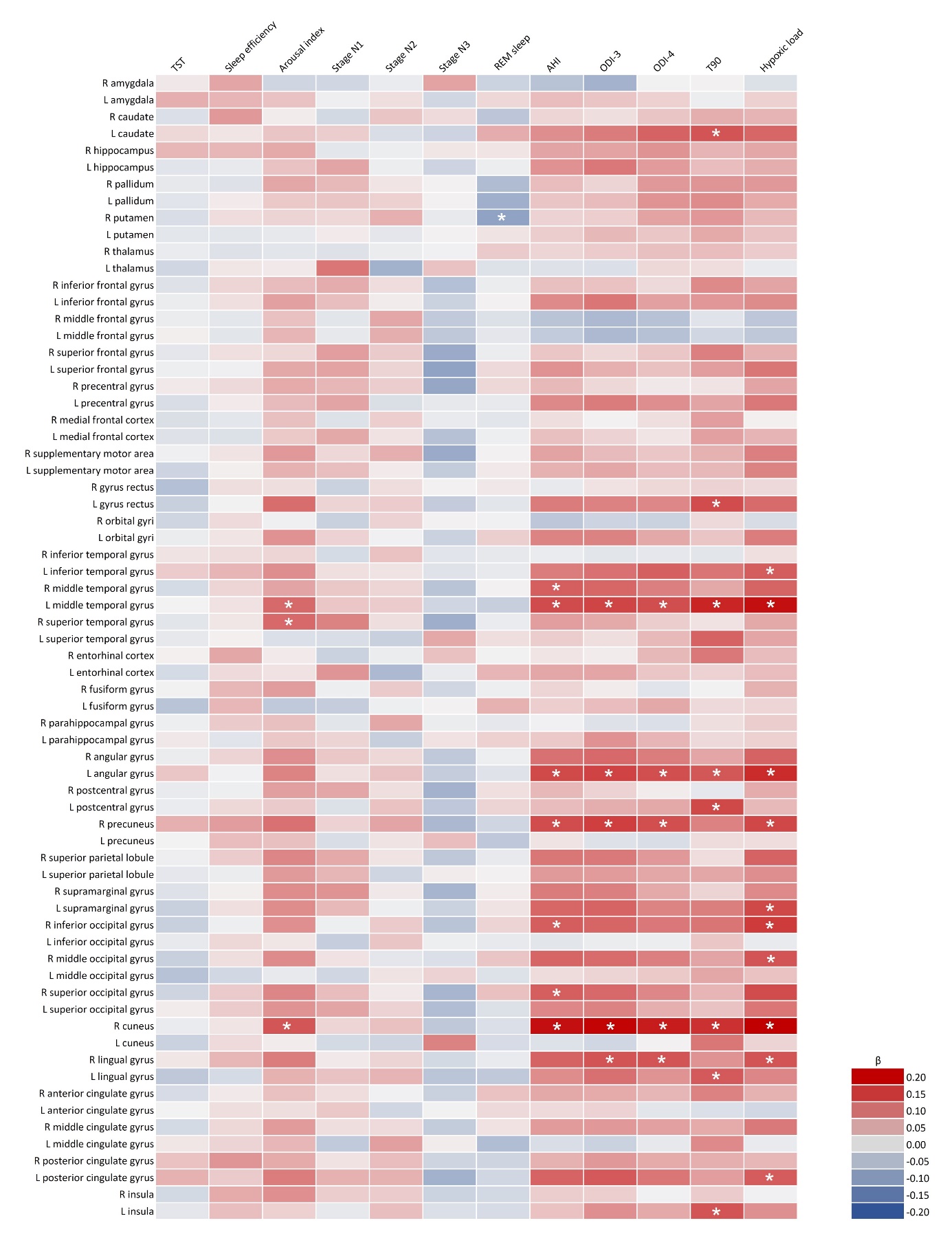
**

**Supplementary Table 1. Differences in R2* among obstructive sleep apnea groups.** Data are seconds^-1^ and are presented as adjusted means ± standard errors. Data are analyzed by analysis of covariance using R2* as dependent variable and obstructive sleep apnea groups as independent variable. Models are adjusted for age, time between polysomnography and MRI, sex, body mass index, diabetes, dyslipidemia, hypertension, smoking, and continuous positive airway pressure treatment. Bold text indicates p-value < 0.005. Abbreviations: ^a^ = significant difference compared to no OSA group; ^b^ = significant difference compared to mild OSA group; ^c^ = significant difference compared to moderate OSA group; L = left; R = right.

|  | **No OSA** | **Mild OSA** | **Moderate OSA** | **Severe OSA** | **P-value** |
| --- | --- | --- | --- | --- | --- |
|  | **n=241 (31.2%)** | **n=286 (37.0%)** | **n=164 (21.2%)** | **n=82 (10.6%)** |  |
| R amygdala | 14.97 ± 0.23 | 14.79 ± 0.23 | 14.66 ± 0.23 | 14.65 ± 0.26 | 0.405 |
| L amygdala | 17.35 ± 0.18 | 17.53 ± 0.18 | 17.69 ± 0.18 | 17.53 ± 0.20 | 0.165 |
| R caudate | 23.98 ± 0.37 | 24.10 ± 0.36 | 24.24 ± 0.36 | 24.44 ± 0.41 | 0.684 |
| L caudate | 18.47 ± 0.15 | 18.59 ± 0.15 | 18.65 ± 0.15 | 18.81 ± 0.16 | 0.194 |
| R hippocampus | 15.75 ± 0.16 | 15.86 ± 0.16 | 15.92 ± 0.16 | 16.05 ± 0.18 | 0.380 |
| L hippocampus | 18.52 ± 0.52 | 18.74 ± 0.51 | 19.11 ± 0.51 | 18.84 ± 0.58 | 0.603 |
| R pallidum | 37.35 ± 0.64 | 36.90 ± 0.62 | 37.52 ± 0.63 | 39.06 ± 0.70 | 0.007 |
| L pallidum | 29.32 ± 0.61 | 29.65 ± 0.59 | 29.87 ± 0.59 | 30.99 ± 0.67 | 0.082 |
| R putamen | 29.42 ± 0.59 | 29.59 ± 0.58 | 29.87 ± 0.58 | 30.84 ± 0.65 | 0.143 |
| L putamen | 19.83 ± 0.21 | 20.00 ± 0.20 | 20.08 ± 0.20 | 20.14 ± 0.23 | 0.424 |
| R thalamus | 20.24 ± 0.22 | 20.39 ± 0.21 | 20.44 ± 0.21 | 20.51 ± 0.24 | 0.575 |
| L thalamus | 21.61 ± 0.24 | 21.70 ± 0.24 | 21.69 ± 0.24 | 21.70 ± 0.27 | 0.953 |
| R inferior frontal gyrus | 17.63 ± 0.12 | 17.67 ± 0.12 | 17.58 ± 0.12 | 17.94 ± 0.13 | 0.022 |
| L inferior frontal gyrus | 17.90 ± 0.12 | 18.06 ± 0.12 | 18.09 ± 0.12 | 18.14 ± 0.14 | 0.140 |
| R middle frontal gyrus | 17.33 ± 0.37 | 16.94 ± 0.36 | 17.01 ± 0.36 | 17.10 ± 0.41 | 0.445 |
| L middle frontal gyrus | 17.61 ± 0.43 | 17.10 ± 0.42 | 17.19 ± 0.42 | 17.32 ± 0.48 | 0.336 |
| R superior frontal gyrus | 17.48 ± 0.11 | 17.49 ± 0.10 | 17.54 ± 0.10 | 17.66 ± 0.12 | 0.397 |
| L superior frontal gyrus | 17.91 ± 0.12 | 17.95 ± 0.12 | 17.94 ± 0.12 | 18.36 ± 0.13^a, b, c^ | **0.003** |
| R precentral gyrus | 19.17 ± 0.14 | 19.21 ± 0.14 | 19.15 ± 0.14 | 19.48 ± 0.16 | 0.116 |
| L precentral gyrus | 18.71 ± 0.15 | 18.86 ± 0.14 | 18.80 ± 0.14 | 19.02 ± 0.16 | 0.182 |
| R medial frontal cortex | 17.07 ± 0.16 | 17.16 ± 0.16 | 16.93 ± 0.16 | 17.25 ± 0.18 | 0.145 |
| L medial frontal cortex | 17.40 ± 0.11 | 17.39 ± 0.11 | 17.47 ± 0.11 | 17.64 ± 0.12 | 0.119 |
| R supplementary motor area | 17.89 ± 0.12 | 17.92 ± 0.12 | 17.88 ± 0.12 | 18.28 ± 0.13 | 0.006 |
| L supplementary motor area | 17.19 ± 0.10 | 17.22 ± 0.10 | 17.19 ± 0.10 | 17.43 ± 0.11 | 0.076 |
| R gyrus rectus | 23.89 ± 0.55 | 24.15 ± 0.54 | 23.58 ± 0.54 | 24.43 ± 0.61 | 0.348 |
| L gyrus rectus | 19.91 ± 0.15 | 20.00 ± 0.14 | 20.01 ± 0.14 | 20.43 ± 0.16 | 0.009 |
| R orbital gyri | 21.47 ± 0.29 | 21.59 ± 0.28 | 21.15 ± 0.28 | 21.24 ± 0.32 | 0.194 |
| L orbital gyri | 17.44 ± 0.10 | 17.50 ± 0.10 | 17.43 ± 0.10 | 17.81 ± 0.11^a, b, c^ | **0.002** |
| R inferior temporal gyrus | 21.68 ± 0.26 | 21.81 ± 0.26 | 21.80 ± 0.26 | 21.90 ± 0.29 | 0.840 |
| L inferior temporal gyrus | 19.71 ± 0.16 | 19.92 ± 0.16 | 19.95 ± 0.16 | 20.07 ± 0.18 | 0.126 |
| R middle temporal gyrus | 17.88 ± 0.12 | 18.02 ± 0.12 | 18.02 ± 0.12 | 18.35 ± 0.14^a, b, c^ | **0.004** |
| L middle temporal gyrus | 21.22 ± 0.20 | 21.44 ± 0.19 | 21.54 ± 0.19 | 22.15 ± 0.22^a, b, c^ | **<0.001** |
| R superior temporal gyrus | 17.84 ± 0.12 | 17.89 ± 0.12 | 17.87 ± 0.12 | 18.04 ± 0.13 | 0.423 |
| L superior temporal gyrus | 16.49 ± 0.21 | 16.75 ± 0.20 | 16.51 ± 0.20 | 16.86 ± 0.23 | 0.101 |
| R entorhinal cortex | 15.41 ± 0.20 | 15.34 ± 0.20 | 15.17 ± 0.20 | 15.50 ± 0.22 | 0.324 |
| L entorhinal cortex | 16.45 ± 0.14 | 16.57 ± 0.13 | 16.57 ± 0.13 | 16.64 ± 0.15 | 0.463 |
| R fusiform gyrus | 19.12 ± 0.20 | 19.20 ± 0.19 | 19.21 ± 0.19 | 19.35 ± 0.22 | 0.747 |
| L fusiform gyrus | 25.73 ± 0.69 | 26.82 ± 0.67 | 26.40 ± 0.67 | 25.93 ± 0.76 | 0.093 |
| R parahippocampal gyrus | 18.36 ± 0.28 | 18.33 ± 0.27 | 18.21 ± 0.27 | 18.66 ± 0.31 | 0.441 |
| L parahippocampal gyrus | 15.56 ± 0.16 | 15.71 ± 0.16 | 15.54 ± 0.16 | 15.84 ± 0.18 | 0.158 |
| R angular gyrus | 18.15 ± 0.11 | 18.23 ± 0.11 | 18.26 ± 0.11 | 18.46 ± 0.12 | 0.068 |
| L angular gyrus | 19.60 ± 0.15 | 19.78 ± 0.15 | 19.77 ± 0.15 | 20.24 ± 0.17^a, b, c^ | **0.001** |
| R postcentral gyrus | 18.91 ± 0.13 | 18.92 ± 0.12 | 18.92 ± 0.12 | 19.16 ± 0.14 | 0.220 |
| L postcentral gyrus | 17.60 ± 0.18 | 17.74 ± 0.17 | 17.75 ± 0.17 | 17.78 ± 0.20 | 0.622 |
| R precuneus | 18.01 ± 0.12 | 18.16 ± 0.12 | 18.19 ± 0.12 | 18.42 ± 0.13 | 0.019 |
| L precuneus | 17.64 ± 0.29 | 17.83 ± 0.28 | 17.48 ± 0.28 | 17.86 ± 0.32 | 0.329 |
| R superior parietal lobule | 18.69 ± 0.11 | 18.78 ± 0.11 | 18.80 ± 0.11 | 19.02 ± 0.12 | 0.045 |
| L superior parietal lobule | 17.81 ± 0.12 | 17.93 ± 0.11 | 17.86 ± 0.11 | 18.06 ± 0.13 | 0.154 |
| R supramarginal gyrus | 17.70 ± 0.10 | 17.75 ± 0.10 | 17.79 ± 0.10 | 17.99 ± 0.12 | 0.078 |
| L supramarginal gyrus | 19.94 ± 0.14 | 20.09 ± 0.14 | 20.07 ± 0.14 | 20.50 ± 0.16^a, b, c^ | **0.003** |
| R inferior occipital gyrus | 19.86 ± 0.15 | 20.06 ± 0.14 | 20.04 ± 0.14 | 20.42 ± 0.16 | 0.006 |
| L inferior occipital gyrus | 24.04 ± 0.41 | 24.29 ± 0.40 | 24.01 ± 0.40 | 23.95 ± 0.46 | 0.685 |
| R middle occipital gyrus | 18.74 ± 0.12 | 18.91 ± 0.12 | 18.85 ± 0.12 | 19.12 ± 0.14 | 0.026 |
| L middle occipital gyrus | 20.83 ± 0.37 | 21.36 ± 0.35 | 20.96 ± 0.36 | 21.20 ± 0.40 | 0.140 |
| R superior occipital gyrus | 20.03 ± 0.14 | 20.18 ± 0.14 | 20.16 ± 0.14 | 20.56 ± 0.15 | 0.005 |
| L superior occipital gyrus | 18.36 ± 0.11 | 18.47 ± 0.11 | 18.42 ± 0.11 | 18.70 ± 0.12 | 0.032 |
| R cuneus | 20.19 ± 0.14 | 20.36 ± 0.14 | 20.55 ± 0.14^a^ | 20.84 ± 0.16^a, b^ | **<0.001** |
| L cuneus | 15.74 ± 0.22 | 15.85 ± 0.21 | 15.63 ± 0.22 | 15.86 ± 0.25 | 0.523 |
| R lingual gyrus | 19.71 ± 0.16 | 19.94 ± 0.16 | 20.04 ± 0.16 | 20.09 ± 0.18 | 0.054 |
| L lingual gyrus | 17.94 ± 0.15 | 17.99 ± 0.15 | 17.92 ± 0.15 | 18.43 ± 0.17 | 0.007 |
| R anterior cingulate gyrus | 15.68 ± 0.10 | 15.79 ± 0.10 | 15.74 ± 0.10 | 15.89 ± 0.11 | 0.165 |
| L anterior cingulate gyrus | 15.39 ± 0.12 | 15.51 ± 0.12 | 15.46 ± 0.12 | 15.38 ± 0.14 | 0.451 |
| R middle cingulate gyrus | 16.53 ± 0.11 | 16.62 ± 0.11 | 16.53 ± 0.11 | 16.85 ± 0.12 | 0.019 |
| L middle cingulate gyrus | 17.05 ± 0.19 | 17.05 ± 0.19 | 16.88 ± 0.19 | 17.06 ± 0.21 | 0.619 |
| R posterior cingulate gyrus | 17.72 ± 0.16 | 17.89 ± 0.15 | 17.96 ± 0.16 | 17.85 ± 0.18 | 0.272 |
| L posterior cingulate gyrus | 17.92 ± 0.12 | 18.06 ± 0.12 | 18.06 ± 0.12 | 18.34 ± 0.13 | 0.021 |
| R insula | 15.56 ± 0.11 | 15.66 ± 0.11 | 15.60 ± 0.11 | 15.66 ± 0.12 | 0.583 |
| L insula | 18.06 ± 0.15 | 18.15 ± 0.15 | 17.99 ± 0.15 | 18.29 ± 0.17 | 0.192 |

**Supplementary Table 2. Associations between R2* and sleep architecture variables.** Data are presented as standardized beta coefficient (β). Data are analyzed by linear regression model using R2* as dependent variable and individual sleep variables as independent variables. Models are adjusted for age, time between polysomnography and MRI, sex, body mass index, diabetes, dyslipidemia, hypertension, smoking, and continuous positive airway pressure treatment. Bold text indicates p-value < 0.005. Abbreviations: TST = total sleep time.

|  | **TST** | | **Sleep efficiency** | | **Arousal index** | | **Stage N1** | | **Stage N2** | | **Stage N3** | | **REM sleep** | |
| --- | --- | --- | --- | --- | --- | --- | --- | --- | --- | --- | --- | --- | --- | --- |
|  | **β** | **P-value** | **β** | **P-value** | **β** | **P-value** | **β** | **P-value** | **β** | **P-value** | **β** | **P-value** | **β** | **P-value** |
| R amygdala | 0.009 | 0.798 | 0.063 | 0.114 | -0.036 | 0.355 | -0.033 | 0.400 | -0.008 | 0.827 | 0.066 | 0.083 | -0.039 | 0.296 |
| L amygdala | 0.056 | 0.129 | 0.051 | 0.199 | 0.039 | 0.313 | -0.004 | 0.910 | 0.016 | 0.656 | -0.033 | 0.375 | 0.022 | 0.559 |
| R caudate | -0.023 | 0.517 | 0.075 | 0.049 | 0.006 | 0.881 | -0.031 | 0.400 | 0.038 | 0.287 | 0.020 | 0.580 | -0.055 | 0.119 |
| L caudate | 0.023 | 0.522 | 0.011 | 0.782 | 0.037 | 0.328 | 0.031 | 0.410 | -0.025 | 0.479 | -0.038 | 0.306 | 0.058 | 0.107 |
| R hippocampus | 0.049 | 0.186 | 0.049 | 0.218 | 0.060 | 0.127 | -0.015 | 0.697 | -0.006 | 0.879 | 0.010 | 0.785 | 0.011 | 0.769 |
| L hippocampus | -0.017 | 0.652 | -0.011 | 0.773 | 0.039 | 0.311 | 0.064 | 0.099 | -0.006 | 0.878 | -0.038 | 0.314 | -0.006 | 0.865 |
| R pallidum | -0.012 | 0.755 | -0.020 | 0.608 | 0.062 | 0.113 | 0.047 | 0.229 | 0.011 | 0.766 | 0.002 | 0.951 | -0.068 | 0.066 |
| L pallidum | -0.017 | 0.618 | 0.007 | 0.853 | 0.034 | 0.344 | 0.038 | 0.303 | 0.028 | 0.422 | 0.001 | 0.984 | -0.084 | 0.016 |
| R putamen | -0.026 | 0.461 | 0.018 | 0.636 | 0.025 | 0.488 | 0.023 | 0.524 | 0.055 | 0.110 | -0.011 | 0.759 | -0.099 | **0.004** |
| L putamen | -0.015 | 0.681 | -0.016 | 0.679 | -0.012 | 0.743 | 0.007 | 0.855 | -0.014 | 0.693 | 0.002 | 0.960 | 0.013 | 0.719 |
| R thalamus | -0.008 | 0.819 | -0.020 | 0.603 | -0.016 | 0.662 | -0.020 | 0.593 | -0.007 | 0.838 | -0.002 | 0.956 | 0.033 | 0.349 |
| L thalamus | -0.033 | 0.371 | 0.010 | 0.800 | 0.008 | 0.844 | 0.103 | 0.008 | -0.082 | 0.027 | 0.039 | 0.307 | -0.021 | 0.572 |
| R inferior frontal gyrus | -0.020 | 0.586 | 0.024 | 0.538 | 0.045 | 0.246 | 0.058 | 0.130 | 0.018 | 0.615 | -0.063 | 0.093 | -0.007 | 0.848 |
| L inferior frontal gyrus | -0.007 | 0.843 | 0.015 | 0.708 | 0.067 | 0.085 | 0.043 | 0.266 | 0.005 | 0.890 | -0.041 | 0.283 | 0.001 | 0.978 |
| R middle frontal gyrus | -0.007 | 0.862 | -0.015 | 0.718 | 0.041 | 0.317 | -0.016 | 0.705 | 0.062 | 0.112 | -0.050 | 0.215 | -0.022 | 0.582 |
| L middle frontal gyrus | 0.004 | 0.917 | -0.016 | 0.705 | 0.051 | 0.216 | -0.012 | 0.778 | 0.057 | 0.146 | -0.053 | 0.192 | -0.014 | 0.724 |
| R superior frontal gyrus | -0.014 | 0.708 | 0.015 | 0.709 | 0.023 | 0.544 | 0.070 | 0.067 | 0.033 | 0.363 | -0.090 | 0.015 | -0.007 | 0.851 |
| L superior frontal gyrus | -0.005 | 0.889 | -0.003 | 0.938 | 0.061 | 0.118 | 0.066 | 0.088 | 0.025 | 0.503 | -0.099 | 0.009 | 0.021 | 0.581 |
| R precentral gyrus | 0.008 | 0.820 | 0.021 | 0.590 | 0.059 | 0.126 | 0.049 | 0.212 | 0.031 | 0.399 | -0.094 | 0.012 | 0.023 | 0.538 |
| L precentral gyrus | -0.026 | 0.471 | 0.007 | 0.855 | 0.046 | 0.235 | 0.065 | 0.092 | -0.024 | 0.518 | -0.012 | 0.742 | -0.011 | 0.769 |
| R medial frontal cortex | -0.024 | 0.513 | -0.015 | 0.703 | 0.042 | 0.286 | -0.021 | 0.599 | 0.030 | 0.419 | -0.009 | 0.806 | -0.016 | 0.672 |
| L medial frontal cortex | -0.020 | 0.588 | -0.022 | 0.578 | 0.034 | 0.379 | 0.061 | 0.114 | 0.014 | 0.704 | -0.061 | 0.102 | -0.005 | 0.898 |
| R supplementary motor area | -0.003 | 0.930 | 0.012 | 0.756 | 0.074 | 0.057 | 0.022 | 0.568 | 0.056 | 0.125 | -0.090 | 0.017 | 0.002 | 0.955 |
| L supplementary motor area | -0.038 | 0.299 | 0.004 | 0.924 | 0.053 | 0.166 | 0.043 | 0.259 | 0.007 | 0.852 | -0.048 | 0.193 | 0.008 | 0.823 |
| R gyrus rectus | -0.063 | 0.084 | 0.016 | 0.695 | 0.016 | 0.687 | -0.040 | 0.304 | 0.018 | 0.630 | 0.001 | 0.980 | 0.009 | 0.800 |
| L gyrus rectus | -0.043 | 0.227 | -0.001 | 0.983 | 0.110 | 0.003 | 0.025 | 0.502 | 0.032 | 0.369 | -0.047 | 0.192 | -0.016 | 0.663 |
| R orbital gyri | -0.036 | 0.324 | 0.020 | 0.614 | -0.003 | 0.935 | -0.042 | 0.269 | 0.026 | 0.477 | 0.002 | 0.965 | -0.002 | 0.958 |
| L orbital gyri | -0.007 | 0.844 | 0.011 | 0.784 | 0.080 | 0.037 | 0.027 | 0.488 | -0.002 | 0.960 | -0.041 | 0.272 | 0.029 | 0.431 |
| R inferior temporal gyrus | 0.012 | 0.754 | 0.019 | 0.634 | 0.024 | 0.532 | -0.029 | 0.453 | 0.040 | 0.285 | -0.017 | 0.635 | -0.012 | 0.749 |
| L inferior temporal gyrus | 0.032 | 0.374 | 0.051 | 0.196 | 0.083 | 0.031 | 0.015 | 0.694 | 0.013 | 0.724 | -0.019 | 0.605 | -0.011 | 0.763 |
| R middle temporal gyrus | -0.029 | 0.421 | 0.024 | 0.527 | 0.065 | 0.084 | 0.035 | 0.359 | 0.024 | 0.508 | -0.058 | 0.114 | 0.002 | 0.954 |
| L middle temporal gyrus | 0.000 | 0.993 | 0.014 | 0.718 | 0.113 | **0.004** | 0.036 | 0.355 | 0.032 | 0.377 | -0.035 | 0.359 | -0.044 | 0.238 |
| R superior temporal gyrus | -0.016 | 0.655 | 0.010 | 0.806 | 0.114 | **0.003** | 0.092 | 0.017 | 0.016 | 0.658 | -0.085 | 0.023 | -0.008 | 0.821 |
| L superior temporal gyrus | -0.012 | 0.746 | 0.003 | 0.946 | -0.023 | 0.560 | -0.025 | 0.512 | -0.043 | 0.237 | 0.061 | 0.104 | 0.017 | 0.647 |
| R entorhinal cortex | 0.003 | 0.935 | 0.062 | 0.115 | 0.007 | 0.858 | -0.040 | 0.296 | -0.007 | 0.843 | 0.041 | 0.275 | -0.001 | 0.984 |
| L entorhinal cortex | -0.029 | 0.423 | 0.020 | 0.616 | 0.014 | 0.710 | 0.077 | 0.047 | -0.072 | 0.049 | -0.009 | 0.819 | 0.051 | 0.169 |
| R fusiform gyrus | -0.001 | 0.983 | 0.049 | 0.220 | 0.071 | 0.068 | -0.005 | 0.889 | 0.033 | 0.371 | -0.033 | 0.384 | -0.005 | 0.889 |
| L fusiform gyrus | -0.057 | 0.108 | 0.049 | 0.198 | -0.051 | 0.171 | -0.046 | 0.213 | -0.005 | 0.881 | 0.001 | 0.977 | 0.053 | 0.135 |
| R parahippocampal gyrus | -0.005 | 0.894 | 0.033 | 0.413 | 0.042 | 0.289 | -0.016 | 0.687 | 0.062 | 0.535 | -0.007 | 0.859 | -0.013 | 0.738 |
| L parahippocampal gyrus | 0.009 | 0.806 | -0.017 | 0.665 | 0.035 | 0.365 | 0.025 | 0.527 | -0.044 | 0.238 | 0.014 | 0.711 | 0.028 | 0.453 |
| R angular gyrus | -0.007 | 0.856 | 0.025 | 0.522 | 0.083 | 0.029 | 0.035 | 0.358 | 0.022 | 0.539 | -0.051 | 0.171 | -0.005 | 0.880 |
| L angular gyrus | 0.036 | 0.329 | -0.002 | 0.953 | 0.091 | 0.017 | 0.015 | 0.698 | 0.039 | 0.283 | -0.046 | 0.222 | -0.019 | 0.597 |
| R postcentral gyrus | -0.009 | 0.801 | -0.005 | 0.898 | 0.069 | 0.068 | 0.062 | 0.101 | 0.016 | 0.655 | -0.080 | 0.030 | 0.015 | 0.685 |
| L postcentral gyrus | -0.008 | 0.829 | 0.022 | 0.585 | 0.033 | 0.395 | -0.022 | 0.565 | 0.040 | 0.277 | -0.052 | 0.168 | 0.025 | 0.499 |
| R precuneus | 0.055 | 0.128 | 0.071 | 0.070 | 0.107 | 0.005 | 0.026 | 0.505 | 0.065 | 0.076 | -0.073 | 0.052 | -0.036 | 0.322 |
| L precuneus | 0.000 | 0.999 | 0.044 | 0.272 | 0.042 | 0.279 | -0.025 | 0.531 | 0.013 | 0.727 | 0.044 | 0.255 | -0.052 | 0.163 |
| R superior parietal lobule | -0.004 | 0.903 | 0.032 | 0.408 | 0.089 | 0.019 | 0.060 | 0.114 | 0.012 | 0.744 | -0.053 | 0.153 | -0.011 | 0.760 |
| L superior parietal lobule | -0.013 | 0.720 | -0.007 | 0.860 | 0.073 | 0.053 | 0.050 | 0.189 | -0.035 | 0.326 | 0.005 | 0.893 | 0.001 | 0.980 |
| R supramarginal gyrus | -0.020 | 0.579 | 0.004 | 0.923 | 0.084 | 0.027 | 0.079 | 0.038 | 0.009 | 0.795 | -0.077 | 0.037 | 0.005 | 0.888 |
| L supramarginal gyrus | -0.043 | 0.240 | 0.015 | 0.702 | 0.084 | 0.027 | 0.047 | 0.217 | -0.005 | 0.883 | -0.040 | 0.287 | 0.013 | 0.731 |
| R inferior occipital gyrus | -0.043 | 0.219 | -0.005 | 0.904 | 0.082 | 0.027 | -0.005 | 0.888 | 0.033 | 0.352 | -0.057 | 0.117 | 0.025 | 0.482 |
| L inferior occipital gyrus | -0.011 | 0.758 | 0.023 | 0.573 | 0.010 | 0.807 | -0.045 | 0.253 | 0.038 | 0.303 | -0.005 | 0.905 | -0.011 | 0.763 |
| R middle occipital gyrus | -0.043 | 0.231 | 0.016 | 0.686 | 0.085 | 0.023 | 0.014 | 0.703 | -0.014 | 0.697 | -0.023 | 0.526 | 0.038 | 0.287 |
| L middle occipital gyrus | -0.060 | 0.100 | -0.032 | 0.418 | -0.011 | 0.776 | -0.034 | 0.368 | 0.012 | 0.737 | 0.027 | 0.464 | -0.021 | 0.573 |
| R superior occipital gyrus | -0.037 | 0.302 | 0.033 | 0.405 | 0.089 | 0.020 | 0.044 | 0.247 | 0.008 | 0.821 | -0.076 | 0.040 | 0.041 | 0.265 |
| L superior occipital gyrus | 0.006 | 0.878 | 0.032 | 0.420 | 0.079 | 0.039 | 0.065 | 0.092 | 0.026 | 0.472 | -0.066 | 0.075 | -0.022 | 0.558 |
| R cuneus | -0.008 | 0.826 | 0.011 | 0.773 | 0.129 | **0.001** | 0.024 | 0.529 | 0.041 | 0.253 | -0.053 | 0.154 | -0.023 | 0.534 |
| L cuneus | -0.041 | 0.261 | 0.020 | 0.610 | 0.005 | 0.905 | -0.030 | 0.430 | -0.037 | 0.304 | 0.092 | 0.013 | -0.028 | 0.437 |
| R lingual gyrus | 0.010 | 0.793 | 0.049 | 0.215 | 0.097 | 0.011 | 0.008 | 0.827 | 0.025 | 0.492 | -0.031 | 0.404 | -0.009 | 0.810 |
| L lingual gyrus | -0.053 | 0.150 | -0.029 | 0.459 | 0.053 | 0.169 | 0.039 | 0.318 | 0.052 | 0.152 | -0.056 | 0.137 | -0.051 | 0.163 |
| R anterior cingulate gyrus | -0.028 | 0.443 | 0.008 | 0.838 | 0.041 | 0.296 | 0.018 | 0.645 | -0.002 | 0.950 | -0.042 | 0.265 | 0.040 | 0.281 |
| L anterior cingulate gyrus | 0.000 | 0.996 | 0.017 | 0.672 | 0.017 | 0.656 | 0.035 | 0.364 | -0.034 | 0.348 | -0.001 | 0.987 | 0.021 | 0.566 |
| R middle cingulate gyrus | -0.030 | 0.409 | 0.022 | 0.569 | 0.072 | 0.061 | 0.016 | 0.683 | 0.020 | 0.588 | -0.048 | 0.203 | 0.014 | 0.714 |
| L middle cingulate gyrus | 0.010 | 0.788 | 0.025 | 0.535 | 0.049 | 0.215 | -0.050 | 0.200 | 0.068 | 0.066 | 0.005 | 0.896 | -0.067 | 0.075 |
| R posterior cingulate gyrus | 0.040 | 0.279 | 0.077 | 0.053 | 0.059 | 0.130 | 0.014 | 0.722 | 0.044 | 0.237 | -0.037 | 0.332 | -0.037 | 0.321 |
| L posterior cingulate gyrus | 0.053 | 0.144 | 0.033 | 0.397 | 0.097 | 0.011 | 0.053 | 0.172 | 0.054 | 0.136 | -0.082 | 0.028 | -0.034 | 0.350 |
| R insula | -0.031 | 0.397 | 0.059 | 0.138 | 0.082 | 0.034 | 0.031 | 0.433 | 0.032 | 0.393 | -0.039 | 0.301 | -0.031 | 0.409 |
| L insula | -0.014 | 0.706 | 0.043 | 0.276 | 0.028 | 0.480 | -0.012 | 0.753 | 0.043 | 0.248 | -0.023 | 0.553 | -0.028 | 0.456 |

**Supplementary Table 3. Associations between R2* and sleep respiratory variables.** Data are presented as standardized beta coefficient (β). Data are analyzed by linear regression model using R2* as dependent variable and individual sleep variables as independent variables. Models are adjusted for age, time between polysomnography and MRI, sex, body mass index, diabetes, dyslipidemia, hypertension, smoking, and continuous positive airway pressure treatment. Bold text indicates p-value < 0.005. Abbreviations: AHI = apnea-hypopnea index; ODI = oxygen desaturation index; T90 = percentage of sleep time with oxygen saturation < 90%.

|  | **AHI** | | **ODI-3** | | **ODI-4** | | **T90** | | **Hypoxic load** | |
| --- | --- | --- | --- | --- | --- | --- | --- | --- | --- | --- |
|  | **β** | **P-value** | **β** | **P-value** | **β** | **P-value** | **β** | **P-value** | **β** | **P-value** |
| R amygdala | -0.059 | 0.182 | -0.080 | 0.076 | -0.001 | 0.989 | 0.002 | 0.967 | -0.022 | 0.617 |
| L amygdala | 0.044 | 0.316 | 0.037 | 0.412 | 0.028 | 0.516 | -0.006 | 0.896 | 0.028 | 0.522 |
| R caudate | 0.024 | 0.565 | 0.016 | 0.705 | 0.037 | 0.379 | 0.057 | 0.172 | 0.052 | 0.210 |
| L caudate | 0.083 | 0.053 | 0.098 | 0.025 | 0.119 | 0.005 | 0.131 | **0.002** | 0.115 | 0.007 |
| R hippocampus | 0.065 | 0.142 | 0.066 | 0.144 | 0.079 | 0.072 | 0.052 | 0.230 | 0.062 | 0.155 |
| L hippocampus | 0.080 | 0.069 | 0.101 | 0.024 | 0.072 | 0.100 | 0.044 | 0.303 | 0.057 | 0.190 |
| R pallidum | 0.044 | 0.319 | 0.025 | 0.588 | 0.074 | 0.094 | 0.077 | 0.076 | 0.075 | 0.086 |
| L pallidum | 0.037 | 0.369 | 0.045 | 0.289 | 0.078 | 0.057 | 0.085 | 0.035 | 0.059 | 0.152 |
| R putamen | 0.027 | 0.517 | 0.030 | 0.485 | 0.066 | 0.111 | 0.076 | 0.061 | 0.047 | 0.265 |
| L putamen | 0.031 | 0.463 | 0.048 | 0.264 | 0.036 | 0.390 | 0.060 | 0.145 | 0.039 | 0.355 |
| R thalamus | 0.024 | 0.573 | 0.032 | 0.465 | 0.042 | 0.321 | 0.044 | 0.285 | 0.032 | 0.444 |
| L thalamus | -0.020 | 0.650 | -0.023 | 0.616 | 0.022 | 0.611 | 0.018 | 0.685 | -0.009 | 0.830 |
| R inferior frontal gyrus | 0.042 | 0.340 | 0.040 | 0.371 | 0.021 | 0.622 | 0.086 | 0.044 | 0.065 | 0.133 |
| L inferior frontal gyrus | 0.086 | 0.051 | 0.103 | 0.021 | 0.067 | 0.128 | 0.076 | 0.078 | 0.085 | 0.049 |
| R middle frontal gyrus | -0.058 | 0.212 | -0.072 | 0.128 | -0.060 | 0.190 | -0.018 | 0.689 | -0.054 | 0.235 |
| L middle frontal gyrus | -0.049 | 0.294 | -0.071 | 0.136 | -0.061 | 0.187 | -0.033 | 0.458 | -0.053 | 0.248 |
| R superior frontal gyrus | 0.036 | 0.408 | 0.022 | 0.622 | 0.036 | 0.398 | 0.095 | 0.024 | 0.054 | 0.207 |
| L superior frontal gyrus | 0.080 | 0.069 | 0.055 | 0.219 | 0.041 | 0.352 | 0.070 | 0.102 | 0.102 | 0.019 |
| R precentral gyrus | 0.047 | 0.289 | 0.022 | 0.627 | 0.009 | 0.838 | 0.013 | 0.764 | 0.064 | 0.142 |
| L precentral gyrus | 0.087 | 0.046 | 0.098 | 0.029 | 0.083 | 0.056 | 0.065 | 0.129 | 0.101 | 0.020 |
| R medial frontal cortex | 0.016 | 0.722 | 0.001 | 0.977 | 0.016 | 0.719 | 0.070 | 0.107 | 0.003 | 0.950 |
| L medial frontal cortex | 0.042 | 0.336 | 0.026 | 0.564 | 0.011 | 0.792 | 0.067 | 0.119 | 0.052 | 0.232 |
| R supplementary motor area | 0.066 | 0.131 | 0.046 | 0.306 | 0.036 | 0.413 | 0.054 | 0.207 | 0.095 | 0.028 |
| L supplementary motor area | 0.054 | 0.207 | 0.062 | 0.158 | 0.045 | 0.298 | 0.048 | 0.252 | 0.091 | 0.032 |
| R gyrus rectus | -0.011 | 0.807 | 0.014 | 0.759 | 0.023 | 0.603 | 0.026 | 0.540 | 0.021 | 0.623 |
| L gyrus rectus | 0.098 | 0.021 | 0.093 | 0.032 | 0.088 | 0.036 | 0.137 | **0.001** | 0.112 | 0.007 |
| R orbital gyri | -0.054 | 0.211 | -0.038 | 0.392 | -0.040 | 0.355 | 0.021 | 0.618 | -0.027 | 0.536 |
| L orbital gyri | 0.091 | 0.035 | 0.092 | 0.037 | 0.061 | 0.158 | 0.038 | 0.376 | 0.097 | 0.023 |
| R inferior temporal gyrus | -0.008 | 0.863 | -0.016 | 0.727 | -0.013 | 0.765 | -0.016 | 0.714 | 0.015 | 0.732 |
| L inferior temporal gyrus | 0.097 | 0.026 | 0.107 | 0.016 | 0.120 | 0.006 | 0.104 | 0.015 | 0.123 | **0.004** |
| R middle temporal gyrus | 0.122 | **0.0045** | 0.115 | 0.009 | 0.095 | 0.026 | 0.061 | 0.146 | 0.117 | 0.006 |
| L middle temporal gyrus | 0.138 | **0.002** | 0.143 | **0.001** | 0.136 | **0.002** | 0.170 | **<0.001** | 0.188 | **<0.001** |
| R superior temporal gyrus | 0.070 | 0.106 | 0.060 | 0.181 | 0.031 | 0.471 | 0.014 | 0.738 | 0.061 | 0.158 |
| L superior temporal gyrus | 0.031 | 0.475 | 0.016 | 0.722 | 0.047 | 0.276 | 0.119 | 0.005 | 0.063 | 0.147 |
| R entorhinal cortex | 0.011 | 0.808 | 0.006 | 0.896 | 0.048 | 0.269 | 0.101 | 0.017 | 0.045 | 0.297 |
| L entorhinal cortex | 0.062 | 0.160 | 0.063 | 0.161 | 0.036 | 0.413 | 0.027 | 0.532 | 0.044 | 0.310 |
| R fusiform gyrus | 0.024 | 0.594 | 0.006 | 0.899 | -0.017 | 0.700 | -0.001 | 0.986 | 0.054 | 0.220 |
| L fusiform gyrus | 0.030 | 0.479 | 0.044 | 0.311 | 0.061 | 0.147 | 0.021 | 0.615 | 0.024 | 0.563 |
| R parahippocampal gyrus | -0.002 | 0.968 | -0.020 | 0.668 | -0.023 | 0.602 | 0.017 | 0.689 | 0.031 | 0.486 |
| L parahippocampal gyrus | 0.034 | 0.447 | 0.080 | 0.078 | 0.051 | 0.250 | 0.021 | 0.630 | 0.028 | 0.516 |
| R angular gyrus | 0.106 | 0.013 | 0.114 | 0.009 | 0.097 | 0.023 | 0.063 | 0.132 | 0.119 | 0.005 |
| L angular gyrus | 0.139 | **0.001** | 0.145 | **0.001** | 0.135 | **0.002** | 0.128 | **0.003** | 0.164 | **<0.001** |
| R postcentral gyrus | 0.048 | 0.264 | 0.027 | 0.544 | 0.005 | 0.914 | -0.013 | 0.760 | 0.058 | 0.172 |
| L postcentral gyrus | 0.045 | 0.312 | 0.057 | 0.204 | 0.061 | 0.168 | 0.140 | **0.001** | 0.047 | 0.281 |
| R precuneus | 0.137 | **0.002** | 0.148 | **0.001** | 0.135 | **0.002** | 0.093 | 0.030 | 0.141 | **0.001** |
| L precuneus | 0.013 | 0.764 | -0.020 | 0.667 | -0.026 | 0.550 | 0.019 | 0.667 | 0.018 | 0.688 |
| R superior parietal lobule | 0.102 | 0.018 | 0.100 | 0.022 | 0.075 | 0.079 | 0.018 | 0.670 | 0.119 | 0.005 |
| L superior parietal lobule | 0.073 | 0.087 | 0.072 | 0.101 | 0.062 | 0.147 | 0.062 | 0.143 | 0.083 | 0.051 |
| R supramarginal gyrus | 0.098 | 0.022 | 0.095 | 0.031 | 0.060 | 0.160 | 0.025 | 0.551 | 0.086 | 0.042 |
| L supramarginal gyrus | 0.115 | 0.008 | 0.118 | 0.008 | 0.091 | 0.033 | 0.096 | 0.023 | 0.140 | **0.001** |
| R inferior occipital gyrus | 0.123 | **0.003** | 0.116 | 0.007 | 0.101 | 0.015 | 0.104 | 0.012 | 0.152 | **<0.001** |
| L inferior occipital gyrus | -0.016 | 0.712 | -0.007 | 0.881 | -0.006 | 0.897 | 0.036 | 0.407 | -0.010 | 0.829 |
| R middle occipital gyrus | 0.116 | 0.006 | 0.111 | 0.011 | 0.095 | 0.026 | 0.065 | 0.117 | 0.130 | **0.002** |
| L middle occipital gyrus | 0.019 | 0.658 | 0.025 | 0.568 | 0.034 | 0.432 | 0.062 | 0.143 | 0.040 | 0.356 |
| R superior occipital gyrus | 0.122 | **0.0048** | 0.114 | 0.010 | 0.088 | 0.041 | 0.051 | 0.232 | 0.137 | **0.001** |
| L superior occipital gyrus | 0.088 | 0.042 | 0.092 | 0.039 | 0.056 | 0.194 | 0.039 | 0.357 | 0.103 | 0.017 |
| R cuneus | 0.188 | **<0.001** | 0.197 | **<0.001** | 0.177 | **<0.001** | 0.160 | **<0.001** | 0.213 | **<0.001** |
| L cuneus | -0.021 | 0.631 | -0.033 | 0.459 | 0.001 | 0.989 | 0.102 | 0.015 | 0.025 | 0.565 |
| R lingual gyrus | 0.119 | 0.006 | 0.131 | **0.003** | 0.131 | **0.003** | 0.078 | 0.067 | 0.130 | **0.003** |
| L lingual gyrus | 0.084 | 0.055 | 0.108 | 0.016 | 0.079 | 0.070 | 0.126 | **0.003** | 0.089 | 0.039 |
| R anterior cingulate gyrus | 0.061 | 0.166 | 0.059 | 0.186 | 0.050 | 0.254 | 0.093 | 0.030 | 0.053 | 0.222 |
| L anterior cingulate gyrus | 0.020 | 0.655 | 0.027 | 0.551 | -0.016 | 0.721 | -0.023 | 0.588 | -0.032 | 0.467 |
| R middle cingulate gyrus | 0.072 | 0.098 | 0.075 | 0.093 | 0.067 | 0.125 | 0.059 | 0.170 | 0.100 | 0.021 |
| L middle cingulate gyrus | -0.025 | 0.569 | -0.040 | 0.381 | -0.012 | 0.790 | 0.086 | 0.047 | -0.010 | 0.823 |
| R posterior cingulate gyrus | 0.053 | 0.233 | 0.074 | 0.102 | 0.059 | 0.179 | 0.056 | 0.194 | 0.049 | 0.264 |
| L posterior cingulate gyrus | 0.118 | 0.007 | 0.126 | 0.005 | 0.103 | 0.018 | 0.070 | 0.102 | 0.124 | **0.004** |
| R insula | 0.031 | 0.480 | 0.030 | 0.510 | -0.002 | 0.970 | 0.032 | 0.461 | -0.002 | 0.961 |
| L insula | 0.040 | 0.364 | 0.082 | 0.068 | 0.060 | 0.169 | 0.131 | **0.002** | 0.081 | 0.063 |
